# *Evaluating level of adherence to* nicotine replacement therapy and its impact on smoking cessation: a *systematic review and meta-analysis*

**DOI:** 10.1186/s13690-021-00550-2

**Published:** 2021-03-04

**Authors:** Amanual Getnet Mersha, Parivash Eftekhari, Michelle Bovill, Daniel Nigusse Tollosa, Gillian Sandra Gould

**Affiliations:** 1 School of Medicine, College of Medicine and Health Sciences, University of Gondar, Chechela street, kebele 16, Gondar, Amhara region Ethiopia; 2 School of Medicine and Public Health, The University of Newcastle, University Drive, Callaghan, Newcastle, New South Wales 2308 Australia; 3 Hunter Medical Research Institute, Lot 1, Kookaburra Circuit, New Lambton Heights, Newcastle, NSW 2305 Australia

**Keywords:** Adherence, Meta-analysis, Nicotine replacement therapy, Smoking, Smoking cessation, Systematic review

## Abstract

**Background:**

Nicotine replacement therapy (NRT) has proven effect in assisting smoking cessation. However, its effectiveness varies across studies and population groups. This may be due to differences in the rate of adherence. Hence, this review aims to examine the level of adherence to NRT and to assess if the level of adherence to NRT affects success of smoking cessation.

**Methods:**

A systematic review and meta-analysis was conducted using studies retrieved from five electronic databases (MEDLINE, Scopus, EMBASE, Web of science, and PsycINFO) and grey literature. Pooled analysis was conducted using Stata version 16 software. Methodological quality and risk of bias were assessed using the NIH Quality Assessment Tool. Analyses were done among those studies that used similar measurements to assess level of adherence and successful smoking cessation. Heterogeneity of studies was assessed using the Higgins’ I^2^ statistical test. Funnel plots and Egger’s regression asymmetry test were used to affirm presence of significant publication bias.

**Results:**

A total of 7521 adult participants of 18 years old and above from 16 studies were included in the analysis. Level of adherence to NRT among participants of randomised controlled trials were found to be 61% (95% CI, 54–68%), *p*-value of < 0.001 and I^2^ = 85.5%. Whereas 26% of participants were adherent among participants of population-based studies with 95% CI, 20–32%, *p*-value of < 0.001 and I^2^ = 94.5%. Level of adherence was the lowest among pregnant women (22%) with 95% CI, 18–25%, p-value of 0.31 and I^2^ = 15.8%. Being adherent to NRT doubles the rate of successful quitting (OR = 2.17, 95% CI, 1.34–3.51), p-value of < 0.001 and I^2^ = 77.6%.

**Conclusions:**

This review highlights a low level of adherence to NRT among participants of population-based studies and pregnant women as compared to clinical trials. Moreover, the review illustrated a strong association between adherence and successful smoking cessation. Hence, it is recommended to implement and assess large scale interventions to improve adherence. Health programs and policies are recommended to integrate the issue of adherence to NRT as a core component of smoking cessation interventions.

**Trial registration:**

PROSPERO registration number: CRD42020176749. Registered on 28 April 2020.

**Supplementary Information:**

The online version contains supplementary material available at 10.1186/s13690-021-00550-2.

## Background

Smoking remains the most common preventable cause of chronic diseases [1] and premature mortality [2]. Smoking cessation has shown a considerable effect in improving the health and survival of individuals [3]. Smoking cessation usually requires pharmacotherapy in addition to counseling from health care providers. Nicotine replacement therapy (NRT) is the most commonly utilised smoking cessation medication and it can be administered in the form of transdermal patches, gums, lozenges, sprays, or inhalators. NRT has been accepted as first-line pharmacotherapy for smoking cessation because of its safety and efficacy profile [4]..

Among participates who used NRT as a smoking cessation medication, relatively higher smoking cessation rates were reported in clinical trial participants as compared to participants of population-based studies. For instance, in a 2018 Cochrane review that includes only randomised controlled trials, NRT use increases the rate of successful smoking cessation by 50 to 60% [5]. Whereas, most population-based studies reported a 10 to 30% rise in the rate of successful smoking cessation among individuals who utilised NRT [3, 6, 7]. Variations in success rate may be due to underutilisation or non-proper use of prescribed smoking cessation medications illustrated by some studies conducted in USA and China especially in population-based studies, referred to studies that used data collected from diversified areas of daily life that are outside the scope of highly controlled randomised control trials [8–10]. In general, participants of randomised controlled trials were found to be more likely to take their medications as prescribed by the provider because of additional treatment-related counseling offered by the trial which may resolve their concerns and addressed safety issues. For instance, one study conducted among diabetic patients reported lower adherence rate and medication effectiveness among population-based settings than randomised control participants [11].

A comprehensive literature review on adherence to medications was conducted by the Medication and Compliance Special Interest Group of the International Society for Pharmacoeconomics and Outcomes Research. This review defined adherence as “the extent to which a patient takes treatment in accordance with the prescribed interval and dose of a dosing regimen” [12]. Similarly, the World Health Organisation (WHO) defines adherence as “the extent to which the patient follows medical instructions” [13]. The most important factor affecting the evaluation of adherence and success of quitting is likely to be resuming smoking. It can be controlled by establishing the sequence of non-adherence and relapse or assessing adherence during a pre-specified treatment period and determine abstinence only in those who had been continuously abstinent throughout this specified period [14]. There are inconsistencies across the literature in the definition of adherence to smoking cessation pharmacotherapies. Studies have reported that adherence to NRT is low both within and outside of the context of clinical trials [15].

Studies conducted on medical disorders have demonstrated a strong association between the level of adherence to medications and positive clinical outcomes [16]. Also, consumption of a higher number of gums, lozenges, and inhalers resulted in a better success rate for smoking cessation [9, 10, 17]. Most studies focused on smoking cessation outcomes, rather than on a thorough evaluation of the extent of adherence and its association with smoking cessation.

All in all, the rate of smoking cessation rate was reported to be substantially higher among participants of randomised controlled trials as compared to population-based studies. Most studies conducted in other medical conditions reported a significant association between adherence and treatment outcome. Hence, we hypothesised that this disparity in the success of quitting between study types and specific population groups like pregnant women, where there exists additional perceived concern about the fetal risk of NRT, may partially be explained by a difference in the level of adherence.

Our study aimed to examine the level of adherence to NRT among participants of population-based studies, randomised clinical trials, and during pregnancy. Disparity in the rate of adherence between population-based studies and randomised clinical trials was also evaluated in this review. Moreover, the impact of adherence on the rate of successful smoking cessation was also evaluated. Hence, the findings will inform policymakers and health care providers about the importance of addressing adherence to NRT to improve the rate of successful smoking cessation. These findings can be used to guide researchers to develop interventions that can enhance adherence to NRT.

## Methods

### Study design and search strategies

A systematic review and meta-analysis was conducted according to the PRISMA guidelines [18] and MOOSE for observational studies [19]. The protocol is registered in PROSPERO (registration number CRD42020176749), available from https://www.crd.york.ac.uk/prospero/display_record.php?ID=CRD42020176749. A detailed review protocol was developed before commencing the review. The review protocol was published and it is available from https://bmjopen.bmj.com/content/10/9/e039775. MEDLINE, Scopus, EMBASE, CINAHL, and PsycINFO databases were searched. The initial database search was conducted from the start of indexing to February 25, 2020. Citation alerts were created, and the most recent literature search was updated on July 20, 2020. The search strategy was developed with the assistance of a senior librarian. The free-text words (with truncation) and MeSH terms combined using Boolean logic operators: AND, OR, and NOT. A combination of keywords and phrases like: Smoking, “Smoking cessation”, Cessation, Smoke, Cigarette, Quitting, “Quitting Smoking”, “Medication Adherence”, Adherence, Discontinuation, Compliance, Non-Compliance, Non-adherence, “Treatment Compliance”, “Therapeutic Compliance”, “Nicotine replacement therapy”, NRT, “Nicotine patch”, Patch, “Nicotine gum”, “Nicotine inhaler”, Inhaler, Lozenge, “Nicotine spray”, Pharmacotherapies, “Drug therapies”, “Pharmacological therapy”, and “Medication treatment” were used to search articles in the databases [Supplementary material 1]. References from eligible studies were hand-searched for additional studies. Grey literature searches were conducted at the following websites and organisations: Centres for Disease Control and Prevention Smoking and Health Resource Library, National Institute for Health and Care Excellence, and the Ottawa Heart Institute’s Ottawa Model for Smoking Cessation. Citations were gathered using Endnote reference management software version 9 and exported to Covidence software for screening [20].

### Eligibility criteria

#### Population

Studies that enrolled the adult population (18 years old and above) using NRT to quit smoking were included. Studies restricted to participants with mental illness and individuals with other substance use disorders were excluded from the review to maintain homogeneity among studies.

#### Intervention

The intervention included the use of different treatment durations and doses of NRT products taken in forms of gum, transdermal patch, nasal spray, lozenges, oral spray, or an oral inhalator. Studies using medications other than NRT were excluded from the review.

#### Comparator

In clinical trials, the control was either standard care or placebo, behavioural intervention, or no intervention. Studies that compare the effectiveness of NRT with other smoking cessation medications such as bupropion or varenicline were excluded.

#### Outcome

Studies that reported the level of adherence to NRT and/or impact of adherence on the rate of successful smoking cessation were included. We included studies if they reported both outcomes (level and impact of adherence) or one of the outcomes.

#### Study design

Studies that used quantitative methodology such as case-control, cohort, cross-sectional, longitudinal, randomised control trials without limitation to publication date, sample size, setting, language was included in this review. Commentaries, expert opinion, abstracts, conference presentations without complete results were excluded.

#### Screening and data extraction

Each citation was screened by two authors (AM, DT) by using Covidence [20]. Two authors (AM, DT) independently reviewed the full text. A data extraction template was developed and pretested by extracting data from three articles [8, 21, 22] and necessary modifications made before proceeding with the data extraction. The template had three main sections: study identification, methodological characteristics, main findings of the included studies.

#### Quality assessment

Two authors (AM, DT) independently assessed the quality of studies using the National Institutes of Health (NIH) quality assessment tool for observational and interventional studies [23]. The National Institutes of Health (NIH) quality assessment tool identifies the source of bias and study implementation errors through appraising each study against prespecified items including controlling for confounding factors, study power, the strength of causality in the association between interventions and outcomes. Disagreements were resolved by discussion and mutual agreement between the reviewers. [Supplementary material 2].

### Statistical analysis

Meta-analyses were conducted using Stata software (V16, Stata Corp LP, College Station, TX) [24]. Heterogeneity was assessed using the Higgins’ I^2^ statistical analysis test. Heterogeneity was considered low, moderate, or high when the values were below 25%, between 25 and 75%, and above 75%, respectively [25]. Results were pooled using proportion and odds ratios, 95% confidence intervals calculated with *p* values for each outcome variable. When the level of heterogeneity was low, the Mantel-Haenszel fixed-effect model was applied to pool results. When the I^2^ test is above 75%, the DerSimonian-Laird (DL) random-effects model. Funnel plot test of asymmetry and Egger’s regression asymmetry test with *p-value* < 0.05 was used as a cut-off point to confirm a statistically significant publication bias [26].

To decrease heterogeneity between studies, we analysed randomised control trials (as defined by the Cochrane practice guide as the comparison groups generated by random allocation) [25] and population-based studies [27] separately. Pooled analyses were conducted among those studies that used similar measurements to assess the level of adherence and successful smoking cessation in a follow-up period between four and 10 weeks, as most of the published studies follow up period fell within this time frame. Studies that recruited pregnant women only were analysed alone as perinatal concerns may impose additional effects on NRT consumption and adherence [28].

### Operational definitions

#### Adherence

Adherence is defined by The World Health Organisation (WHO) as “the extent to which the patient follows medical instructions” [13]. There exist inconsistencies in the definition of adherence to smoking cessation medications across studies. Hence, the definitions and measurements used to determine adherence to NRT in each study are presented in a summary table [Table 1].

**Table 1:** Overview of studies included in the systematic review and meta-analysis (*N* = 16)

Source	Participants	Study design and sample size	Intervention	Follow up period	The definition used to assess outcomes (adherence to NRT and successful smoking cessation)	Main outcomes on level and impact of adherence on smoking cessation
Balmford et al., 2010, Australia, USA, UK, Canada, [29]	Smokers or recent quitters who had used medication in the last year	Cross-sectional, 981	Participants provided with NRT for 8 weeks.	Participants were followed up to 6 months	Adherence is defined as the use of NRT for 8 weeks, with those who terminated before this cut point considered to have stopped prematurely. Successful smoking cessation was defined as continuous abstinence at six months.	Among the participants, 71.4% of NRT users discontinued medication use prematurely. Those who discontinued use prematurely were significantly less likely to achieve abstinence than those who completed the course of medication (OR = 0.16, 95% CI = 0.08–0.31).
Ben Taleb et al., 2015, Syria, [30]	Adult smokers 18 to 65 years old	RCT, 269	Participants provided with 6 weeks of nicotine patches.	Participants were followed up to 6 weeks	Participants were asked whether they had followed treatment instructions to use one patch every day over the past week. NRT adherence defined being adherent to patch use as responding “yes” to this question during at least 5 of the 6 weeks (> 80%).	Among participants on the nicotine patch, 68% were found to be adherent to pharmacological treatment.
Berg et al., 2013, USA, [31]	Adult smokers 18 years of age or above	RCT, 202	All participants received nicotine patches.	Participants were followed at 3, 6, and 12 months	Calculated adherence level as the number of patches used (80% adherence as adherent; < 80% adherence was considered nonadherent)	Among the study participants, 66.8% were adherent to the patch.
Bolliger et al., 2000, Switzerland, [32]	Adults 18 years of age and above	RCT, 400	Participants were provided with the inhaler as needed for up to 18 months.	Participants were followed at 1, 2, 3, and 6 weeks.	Adherence is defined as utilisation of inhaler every day. Smoking cessation is defined as a decrease in verified measurement of exhaled carbon monoxide at each time point compared with the measurement at baseline.	At week six, 222/368 (60%) participants used the inhaler every day. At 4, 12, and 18 months 146/318 (46%), 39/331 (12%), and 30/289 (10%) participants respectively used the inhaler every day.
Coleman et al., 2012, UK, [33]	Pregnant women GA 12–24 weeks smoked 5 or more cigarettes per day	RCT, 521	Participants were provided with 8 weeks of nicotine patches (15 mg per 16 h).	Participants were followed up to 8 weeks	Using the nicotine patches daily for at least 1 month.	Only 7.2% of women assigned to nicotine-replacement therapy used patches for more than 1 month
Fish et al., 2009, USA, [34]	Pregnant women, GA 13–25 weeks, smoked at least 100 cigarettes in their lifetime, currently smoking five or more per day	RCT, 104	6 weeks of NRT (choice of patch, gum, or lozenge) provided for the treatment arm. Women who chose the patch were provided with a 7-mg patch for fewer than 10 cigarettes/day, 14-mg patch for 10–14 cigarettes/day, and 21-mg patch for 15+ cigarettes/day. Gum or lozenge users were instructed to use one 2-mg piece for every cigarette smoked per day.	Participants were followed up to 6 weeks	Total days of nicotine patch use per week of NRT use over the 6 weeks treatment period.	29% of the 104 women used NRT for the recommended 6 weeks as directed by the health provider.
Hollands et al., 2013, UK, [21]	Adult smokers starting a quit attempt	RCT, 633	All participants were prescribed a nicotine patch and oral NRT. Those smoking 15 or more cigarettes daily were prescribed 21 mg/24 h patches and those smoking 10–14 cigarettes daily were prescribed 14 mg patches.	Participants were followed up to 4 weeks	The proportion of all NRT prescribed consumed each day, averaged over the 4-week treatment period. Quitting is defined as self-reported abstinence from smoking at 4 weeks.	Participants using > 80% of prescribed NRT over 4 weeks 83.8 (351). Higher consumption of NRT was associated with a nonsignificant increase in abstinence (*p* = .093 each additional mg/day consumed was associated with increased odds of abstinence of 5%. OR = 1.05 (95%CI, 1.01–1.10).
Hotham et al., 2006, Australia, [35]	Smoking at least 15 cigarettes per day, GA 12–28 weeks	RCT, 20	Participants offered nicotine patches (15 mg/16 h) for a maximum of 12 weeks.	Participants were followed up to 12 weeks	Self-reported nicotine patch use as directed by the health care provider for the duration of up to 12 weeks.	Only 5 (25%) women used patches continuously up to the 12-week maximum.
Kapur et al., 2001, Canada, [36]	Pregnant women GA 12–24 weeks, who smoke 15 or more cigarettes per day	RCT, 104	Participants were provided with 18 h patch of nicotine 15 mg for 8 weeks, 10 mg for an additional 2 weeks, and 5 mg for the last 2 weeks.	Participants were followed up to 12 weeks	Self-reported use of NRT as prescribed for the duration of up to 12 weeks.	Among participants, 4 (23.5%) women completed the whole course of prescribed medication
Lam et al., 2004, China, [8]	Adult current smokers	Cross-sectional, 1051	Participants are provided with NRT for 12 weeks.	Participants were followed up to 12 months	Self-reported use of NRT daily for at least 4 weeks during the first 3 months. Quitting is assessed by asking whether the subjects had smoked any cigarette during the past 7 days at the 12 months (point prevalence quit rate).	The prevalence of adherence (self-reported NRT use for at least 4 weeks) was 16% (95% confidence interval 14–18%). The quit rate in the adherent group (40%) was greater than that of the non-adherent group (25%) (*P* < 0.001).
Schneider et al., 2003, Switzerland, [22]	Adult daily smokers who are motivated to quit	Cross-sectional exploratory study, 82	Participants were provided with a nasal spray.	Participants were followed up to at week 2, and every month for 24 months	The pharmacist checked every spry thoroughly with a software program and compared the total record of puffs since the last visit with the weight of the returned nasal spray bottles during the first month. Self-reported continuous abstinence from smoking from the end of the first month to the end of the week 2 and at 1, 2, 3, 4, 6, 9, 12, 15, 18, 21, and 24 months of follow-up, validated by expired-air carbon monoxide.	Among the participants, 80% (29/36) of the failures were low consumers of the nasal spray (0–15 puffs/ day) compared with 54% (25/46) of the abstainers. Only 11% (5/46) of the abstainers and 3% (1/36) of the failures used the spray extensively during the first month of the study (i.e., more than 30 puffs/day).
Shiffman et al., 2008, USA, [37]	Adult current smokers	RCT, 204	A 6-week supply of nicotine patches was provided.	Participants were followed up to 6 weeks	Using the patch for ≥20 of the first 21 days of treatment. Smoking cessation is defined as self-reported abstinence at 6 weeks.	Rates of adherence did not differ significantly between the active and placebo groups (139 [68.1%] and 114 [68.3%], respectively). Among active patch users, the odds of abstinence at 6 weeks were more than 3 times greater for adherent versus non-adherent subjects (53.2% vs 21.5%, respectively; OR = 4.20; 95% CI, 1.51–11.72; *P* = 0.006), (*P* = 0.027).
Shiffman et al., 2006, USA, [10]	Adults who maintained abstinence for the first 2 weeks of treatment.	RCT, 1020	Participants were provided with lozenge and behavioural advice.	Participants were followed up to 6 weeks	Average daily lozenge use during the first 2 weeks of therapy. Smoking cessation assessed as a 28 days continuous abstinence, verified by carbon monoxide readings < 10 ppm.	The odds of smoking cessation were 1.25; CI = 1.05–1.50, *P* < 0.02) higher for the adherence group.
Voci et al., 2016, Canada, [38]	18 years or older who smoked at least 10 cigarettes daily	Cross-sectional, 1605	Participants were provided with NRT. The selection of types of NRT was based on participant preference.	Participants were followed up to 10 weeks	Self-reported amount of NRT used over 10 weeks period. The quit outcome was a 7-day point-prevalence of abstinence at a 6-month follow-up.	Of those with end-of-treatment follow-up data on amount of NRT used (*n* = 1605), 19.8% (*n* = 318) used all 10 weeks of NRT provided. Poor quit success was reported by those who used either some (AOR = 0.43, 95% CI = 0.26–0.69, *p* = 0.001) or none (AOR = 0.30, 95% CI = 0.09–0.95, *p* = 0.041) of the NRT versus all 10 weeks.
Wisborg et al., 2000, Denmark, [39]	Pregnant women who smoked 10 or more cigarettes after the first trimester	RCT, 124	Participants were provided with 15-mg patches (16 h/day) for 8 weeks, and/or 10-mg patches (16 h/day) for 3 weeks.	Participants were followed up to 8 weeks	The number of nicotine patches used in 8 weeks period. Quitting is defined as self-reported abstinence of at least 7 days at second, third, and fourth prenatal visits.	In the nicotine group, 17% used all nicotine patches as prescribed by the health care provider.
Yingst et al., 2015, USA, [40]	current daily smokers	Follow-up cross-sectional study, 201	Participants were provided with NRT for a 2-week supply (14 patches) of 21 mg/24-h nicotine patches at the first group visit.	Participants were followed up to 4 weeks	Adherence to the directed use of the nicotine patch was measured by the number of self-reported days, of 28 days, the patch was worn during the quit attempt in treatment. Participants were considered adherent if the patch was worn all 28 days and non-adherent if the nicotine patch was worn less than 28 days.	Among participants, 71 (35.3%) participants were adherent for the first 28 days of treatment and 130 (64.7%) participants were non-adherent.

#### Abstinence

Abstinence is defined as the proportion of participants who achieved point prevalence abstinence up to a given point of time. The assessment of abstinence is usually based on self-report or measuring a biomarker such as salivary cotinine or exhaled carbon monoxide [41]. The definition and measurements used to define successful smoking cessation are summarised in table one [Table 1].

### Ethics consideration

As this is a literature review of the already published studies, it did not require ethical clearance to analyse published articles.

## Results

### Studies identified

**Fig. 1:**
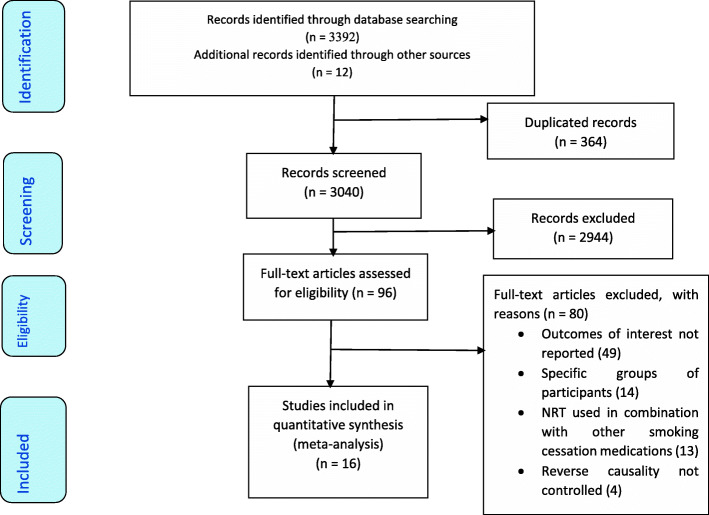
PRISMA Flow diagram of studies included in the review

A total of 3404 articles were identified from five electronic databases and other sources such as grey literature. As illustrated in the flow chart, 16 studies with a total sample size of 7521 participants were included in the systematic review and meta-analysis [Fig. 1].

### Description of the studies

Out of the included 16 studies, 11 studies were clinical trials [10, 21, 30–32, 34–36, 39, 42, 43] and five studies were population-based studies [8, 22, 29, 38, 40]. All of the population-based studies followed a cross-sectional study design [8, 22, 29, 38, 40]. Five studies were conducted in the USA [10, 31, 34, 37, 40]; two studies were conducted in Switzerland [22, 32]; two studies were from the UK [21, 33]; one study involved participants from four countries (Australia, USA, UK, and Canada) [29]. The remaining six studies were from Canada, Denmark, Australia, Syria, and China [8, 30, 35, 36, 38, 39]. The sample size of studies among studies that recruited general adult population [8, 21, 22, 29–32, 38, 40] ranged from a minimum of 82 individuals [22] to a maximum of 1605 participants [38]. All of the studies conducted among the general adult population evaluated adherence between four to 10 weeks [8, 21, 22, 29–32, 38, 40]. Most of the studies used Russell’s standard of smoking abstinence, the self-reported seven-day point prevalence of abstinence validated by expired-air carbon monoxide level [30], while a few studies used continuous abstinence for the follow-up period as a measure of successful smoking cessation. The specific assessment used to evaluate adherence and success of smoking cessation is illustrated in Table 1. Five studies enrolled only pregnant women and all of the five studies were clinical trials [33–36, 39]. The number of participants, in studies that recruited only pregnant women among those on the active nicotine group, was 20 participants in a study conducted in Australia [35] to a maximum of 521 in a study conducted in the UK [33]. All of the studies conducted among pregnant women enrolled those whose gestational age was in the second trimester and above [Table 1].

### Risk of bias

Generally, almost all of the included studies were assessed to have good quality by both reviewers. More detailed assessments of study qualities are illustrated in Supplementary Table 1. Clinical trials scored from a minimum of 11 [21, 34–36] to a maximum of 14 [32, 42] out of a score of 14 on the NIH quality assessment tool for randomised controlled trials. Whereas, population-based studies scored from a minimum of 9 [22] to a maximum of 13 [8] out of a score of 14 on the NIH Quality Assessment Tool for observational studies. [Supplementary material 2].

### Level of adherence to NRT in clinical trials

**Fig. 2:**
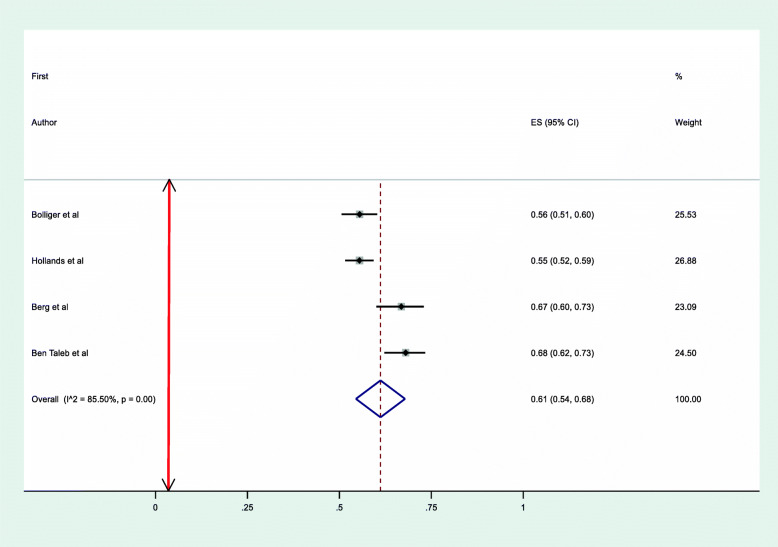
Forest plot illustrating the rate of adherence to NRT in Randomised Controlled Trials

A pooled analysis using random effects model was conducted among clinical trials that assessed level of adherence to NRT for a period of four to ten weeks [21, 30–32] found that 61% (95% CI, 54–68%) of adults met study adherence criteria with a *p*-value of < 0.001 and Higgins’ I^2^ = 85.5% [Fig. 2]. Test of publication bias was examined by using a funnel plot that illustrated the relatively symmetrical distribution of the studies. Furthermore, Egger’s test was conducted and there was no evidence of significant publication bias (*p*-value = 0.093). [Table 2].

**Table 2:**
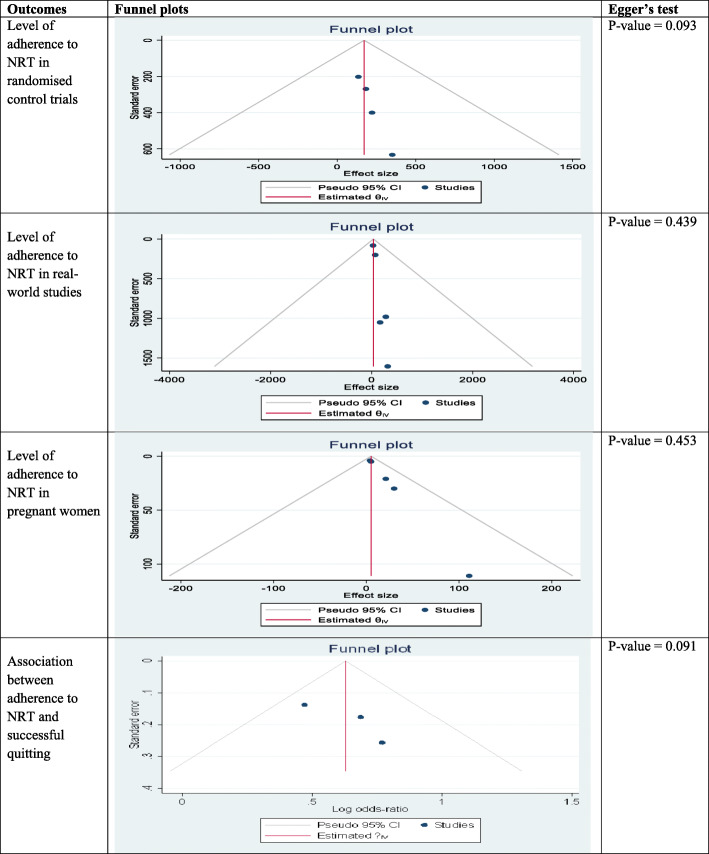
Publication bias of included studies

Among the clinical trials the highest level of adherence of 68% (95% CI, 62–73%) was reported in a study conducted in Syria which assessed adherence by asking participants whether they had followed treatment instructions to use one patch every day over the past week and adherence to patch use was defined as responding “yes” to this question during at least 5 of the 6 weeks (> 80%) [30]. Whereas, the lowest adherence level was from a study conducted in the UK 55% (95% CI, 52–59%) that defined adherence as consumption of at least 80% of the prescribed NRT averaged over 4 weeks treatment period [44] [Table 1].

### Level of adherence to NRT in population-based studies

**Fig. 3:**
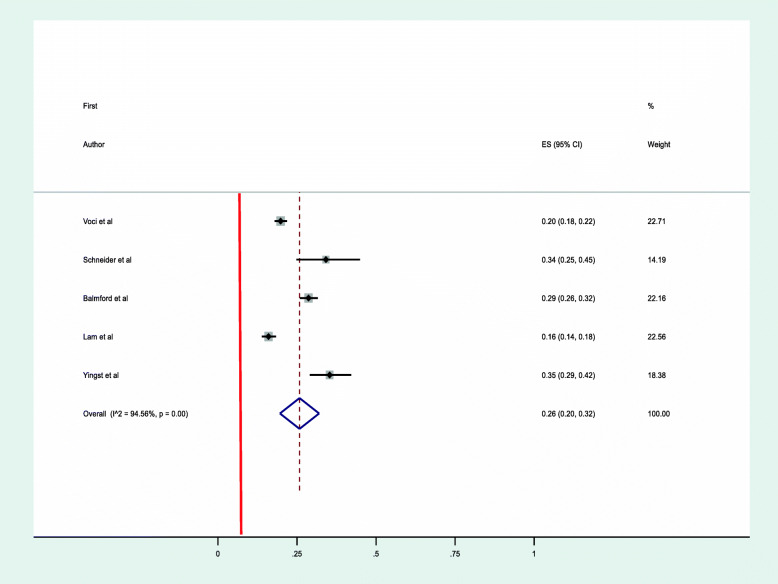
Forest plot illustrating the rate of adherence to NRT in population-based studies

A meta-analysis of five studies [8, 22, 29, 38, 40] that assessed adherence to NRT in population-based studies for a follow-up period between four and 10 weeks among adults was conducted. Using the DerSimonian-Laird random effect model a quarter of participants were found to met the study adherence criteria 26% (95% CI, 20–32%) with a *p*-value of < 0.001 and Higgins’ I^2^ = 94.5%. [Fig. 3]. Funnel plot symmetry test was conducted to evaluate the presence of publication bias and all studies were found to be symmetrical. Egger’s test was also performed, and it demonstrates the absence of significant publication bias (*p*-value = 0.439) [Table 2].

In population-based studies, the extent of adherence ranges from as low as 16% (95% CI, 14–18%) in a study conducted in China [8], that used self-reported daily use of NRT for at least 4 weeks period during 3 months of treatment as a measure of adherence, to a high of 35% (95% CI, 29–42%) in a study from the USA that defined adherence as a self-reported number of days, out of 28 days, that the nicotine patch was worn during the quit attempt. Participants were considered adherent if the patch was worn during all 28 days and non-adherent if the nicotine patch was worn for less than 28 days [Table 1].

### Level of adherence among pregnant women

**Fig. 4:**
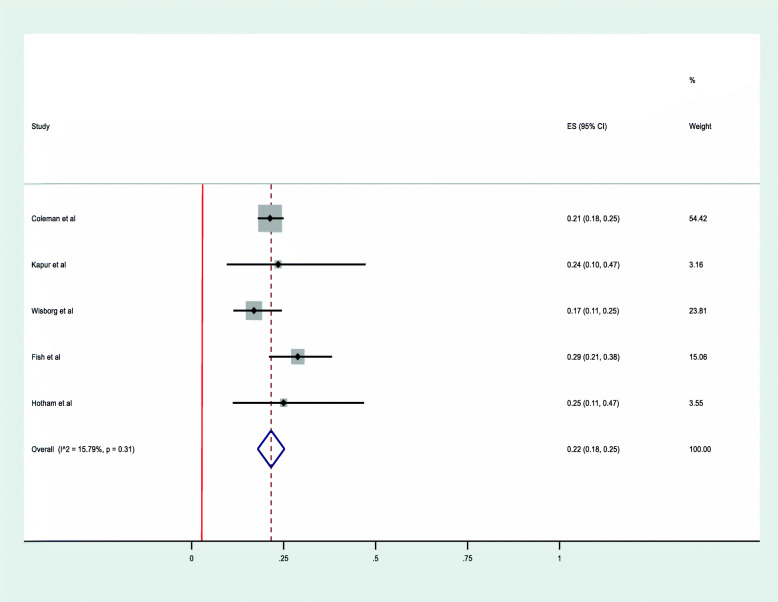
Forest plot illustrating the rate of adherence to NRT among pregnant women

A meta-analysis of five studies [35, 37, 38, 40, 45] assessed pregnant women’s adherence to NRT by using the Mantel-Haenszel fixed-effect model. This illustrated that 22% of pregnant women met the definition of adherence used by the study (95% CI, 18–25%) with a *p*-value of < 0.31 and Higgins’ I^2^ = 15.8% [Fig. 4]. Funnel plot asymmetry test was conducted to evaluate the presence of publication bias and all studies were found to be symmetrical (*p*-value = 0.453) [Table 2]. The rate of adherence was found to be as low as 17% in a study conducted in Denmark [15] to a high of 29% in a study conducted in the USA that used total days of NRT use at delivery to measure the level of adherence [11] [Table 1].

### Association between adherence and successful quitting

**Fig. 5:**
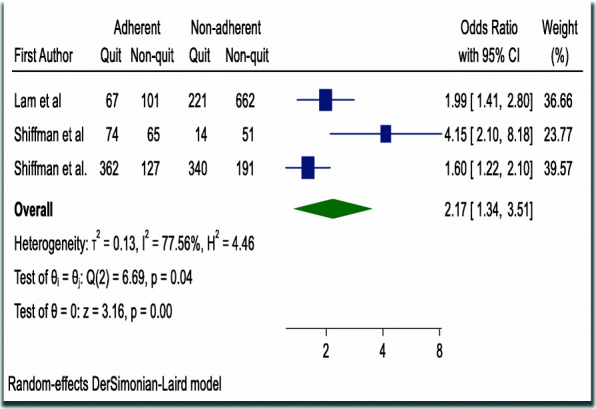
Forest plot illustrating the association between adherence to NRT and smoking cessation

A pooled analysis was done using random effects model among studies that controlled for reverse causality, a potential confounder in assessing adherence and successful smoking cessation [8, 10, 37], illustrated that being adherent to NRT doubled the rate of successful quitting (OR = 2.17, 95% CI 1.34–3.51) with a p-value of < 0.001 and Higgins’ I^2^ = 77.6% [Fig. 5]. Test for small-study effect was examined by using Egger regression-based test and there was no evidence for small study effect in the pooled result. Both visual inspection and formal test for funnel plot asymmetry indicated symmetrical distribution (Egger’s test *P*-value = 0.091) [Table 2].

The strongest association between adherence and successful smoking cessation was observed in a study conducted in the USA which concluded that 21 days of adherence to nicotine patch increases the odds of successful quitting by four-fold [OR = 4.20; 95% CI, 1.51–11.72; *P* = 0.006)] with a significant *P*-value of 0.006 [37] [Table 1].

## Discussion

Overall, the rate of adherence to NRT was found to be more than two folds higher in participants of clinical trials compared to participants of population-based studies. Moreover, only one in five pregnant women were found to be adherent to NRT among clinical trials that enrolled only pregnant women. This review demonstrated that being adherent to NRT doubles the rate of successful smoking cessation among adult daily smokers.

Participants of clinical trials are more motivated to quit smoking and have a better opportunity of obtaining adequate information regarding the safety and efficacy of NRT which could have contributed to the disparity in the level of adherence between participants of trials and population-based studies, as misinformation is a common reason for non-adherence [45, 46]. In a study conducted in the US, misinformation about the safety and efficacy of NRT was linked to a low compliance rate during their smoking cessation attempt. Delivering corrective suggestions was also found to increase awareness and intention to utilise smoking cessation medication s[46]. Randomised controlled trials that incorporate interventions such as counseling with specific emphasis on adherence to NRT and giving information about overcoming challenges to continuing medication use have improved adherence to NRT as well as smoking cessation rates [47, 48]. Compared to non-trials, in clinical trials more attention is given to accomplishing greater medication adherence to NRT [49, 50]. Moreover, participants enrolled in clinical trials may also be more motivated to improve their health, leading to better medication adherence and may differ from the broader population in ways that influence adherence.

The level of adherence was found to be less than a quarter (22%) among pregnant women. This could be due to health professionals’ and women’s concern about the efficacy and safety associated with NRT consumption [51]. Most pregnant women reported safety issues as a cause of non-adherence to NRT, even if evidence showed NRT does not increase the risk of miscarriage, stillbirth, premature birth, low birth weight, admissions to neonatal intensive care, cesarean section, congenital abnormalities or neonatal death [52, 53]. Although NRT is recommended for pregnant women in clinical guidelines in most countries [44, 54, 55], clinicians still hesitate in prescribing NRT during pregnancy [56]. A recent meta-analysis reported a low level of NRT prescription rates for pregnant smokers. Only 25.4% of health care providers reported prescribing NRT for pregnant smokers ever and even very few percentages (6.2%) reported prescribing NRT to pregnant smokers all the time [57]. This hesitancy affects physician counselling concerning adherence and affects clinician-client trust, which has a significant role in medication adherence [58]. Moreover, these reservations regarding the safety of NRT may hamper clinicians to prescribe adequate doses of NRT, as pregnancy is a high metabolic state that requires a relatively higher dose of nicotine to alleviate withdrawal symptoms that may lead to non-adherence [59, 60]. Similarly, a recent review showed the effect of health professionals’ view on the utilisation of NRT. Women feel more confident to use as instructed when the clinician tells NRT is safer than smoking and vice-versa [61].

In this review, adherence to NRT increased the rate of successful smoking cessation by more than two-fold (OR = 2.17, 95% CI 1.34–3.51). This finding is in line with a systematic review conducted in 2013 using clinical trials, which also reported a positive relationship between adherence to NRT and smoking cessation even if it did not compute the magnitude of the impact [62]. Another study assessed the efficacy of nicotine patches and found that consistent use of medications for 3 weeks tripled quitting at the 6 weeks follow up as compared to non-consistent users [43]. Furthermore, interventions aimed at improving adherence to smoking cessation medications raised the rate of short-term and long-term successful smoking cessation [63]. This finding can be explained by the fact that NRT reduces withdrawal symptoms such as craving, depression, restlessness, and irritation by replacing nicotine levels in the bloodstream [30, 64]. Hence, NRT reduces the occurrence of both frequency and strength of urges to smoke [65]. In those participants, who are not taking the medication as prescribed, the extent of withdrawal symptoms will be higher leading to resumed smoking or relapse [65].

### Strength and limitations of the study

This is the first systematic review and meta-analysis to assess the level of adherence to NRT and its impact on the success of smoking cessation. Although pooled analyses were conducted among studies that used relatively similar definitions of adherence, studies that recruited the general adult population, and studies with relatively similar follow up periods, the level of heterogeneity was found to be high. This could be due to a lack of uniform strategies to define and measure adherence to NRT and smoking cessation across the literature. Additional limitation could be excluding studies that compared NRT with other active medications such as Varenicline and Bupropion were not included in the analysis. Hence, caution should be taken in interpreting the findings of this review. The limited number of studies evaluating outcomes and the higher level of heterogeneity among the included studies make it challenging to generate strong conclusions, which should be taken into consideration while using the results of the review.

## Conclusions and recommendations

This review demonstrated the existing low levels of adherence to NRT among adult participants of population-based studies as compared to clinical trial participants. The level of adherence was found to be the lowest among pregnant women enrolled in clinical trials which could be attributed to additional fetal safety concerns. Moreover, this review demonstrated a strong association between the level of adherence to NRT and the success of a smoking cessation attempt.

This review found that smokers participating in clinical trials are more than two times adherent to NRT than participants in population-based studies, which may explain the gap in the effectiveness of the smoking cessation medications between population-based studies and clinical trials highlighted in the literature. This signifies a need for addressing adherence among individuals on smoking cessation medications. Based on the above-mentioned results, it is recommended to improve attention to adherence as a way to potentially improve smoking cessation success. Furthermore, advocating policies and strategies that improve adherence may potentially improve the quality of care an individual receives during his/her quit attempt. Policies and strategies that may enhance the health professionals’ capacity in providing smoking cessation care and adherence to NRT should be advocated. It is also recommended that clinicians support smokers by enhancing their understanding of NRT and supporting them to address their uncertainties about the safety and efficacy of NRT. As pregnancy is found to have a motivational effect to quit smoking, health professionals and researchers are recommended to support and proactively address women’s concerns about nicotine’s fetal and neonatal effects and discuss the relative benefits and harms of continuing to smoke versus a clean safer source of NRT for cessation [28]. Health programs, policies, and activities should incorporate adherence to NRT as a core component of the intervention. Finally, as the area of adherence to NRT is under investigated, future research is recommended to focus on improving adherence at a broader community level as compliance predicts the success of smoking cessation.

## Supplementary Information

**Additional file 1:** Draft Medline search – Ovid interface.

**Additional file 2:** Quality Assessment Tool for observational studies.

## Data Availability

All relevant materials and data supporting the findings of this review are included within the manuscript.

## Abbreviations

*PRISMA*: Preferred Reporting Items for Systematic Reviews and Meta-Analyses
MOOSE: Meta-analyses of observational studies in epidemiology
NRT: Nicotine replacement therapy
