# Supplementary material for: Evaluating level of adherence to nicotine replacement therapy and its impact on smoking cessation: a systematic review and meta-analysis
Source: Arch Public Health. 2021 Mar 4;79:26. doi: 10.1186/s13690-021-00550-2 (PMC7934490; doi:10.1186/s13690-021-00550-2)
Supplement: Supplementary file 1 — Additional file 1: Draft Medline search – Ovid interface. [file 13690_2021_550_MOESM1_ESM.docx]

**Draft Medline search – Ovid interface**

1. (Intervention* or therapy* or treat*). ti,ab.
2. Pharmacotherapy.ti, ab.
3. Nicotine replacement therapy. ti, ab.
4. (Nicotine gums or nicotine patch or nicotine nasal spray or nicotine inhaler or nicotine lozenge). ti,ab.
5. Bupropion. ti,ab.
6. Varenicline .ti,ab.
7. Combination therapy .ti,ab.
8. "Tobacco Use Cessation Products"/
9. (Non-nicotine drug or nicotine receptor partial agonist).ti,ab.
10. 1 or 2 or 3 or 4 or 5 or 6 or 7 or 8 or 9
11. ((quit* or stop* or abstin* or abstain* or reduc* or ceas* or cessation) adj3 (smoke* or tobacco*)).ti,ab.
12. "tobacco use cessation"/ or smoking cessation/
13. 11 or 12
14. 10 AND 13
